# HIV-1 Vpr Functions in Primary CD4^+^ T Cells

**DOI:** 10.3390/v16030420

**Published:** 2024-03-09

**Authors:** Carlos Alberto Vanegas-Torres, Michael Schindler

**Affiliations:** Institute for Medical Virology and Epidemiology of Viral Diseases, University Hospital Tuebingen, 72076 Tuebingen, Germany; carlos.vanegas-torres@med.uni-tuebingen.de

**Keywords:** HIV-1, accessory viral proteins, Vpr, Vif, Nef, Vpu, G2 arrest, CD4^+^ T cells, pathogenesis, inflammation, interferon response, restriction factors

## Abstract

HIV-1 encodes four accesory proteins in addition to its structural and regulatory genes. Uniquely amongst them, Vpr is abundantly present within virions, meaning it is poised to exert various biological effects on the host cell upon delivery. In this way, Vpr contributes towards the establishment of a successful infection, as evidenced by the extent to which HIV-1 depends on this factor to achieve full pathogenicity in vivo. Although HIV infects various cell types in the host organism, CD4^+^ T cells are preferentially targeted since they are highly permissive towards productive infection, concomitantly bringing about the hallmark immune dysfunction that accompanies HIV-1 spread. The last several decades have seen unprecedented progress in unraveling the activities Vpr possesses in the host cell at the molecular scale, increasingly underscoring the importance of this viral component. Nevertheless, it remains controversial whether some of these advances bear in vivo relevance, since commonly employed cellular models significantly differ from primary T lymphocytes. One prominent example is the “established” ability of Vpr to induce G2 cell cycle arrest, with enigmatic physiological relevance in infected primary T lymphocytes. The objective of this review is to present these discoveries in their biological context to illustrate the mechanisms whereby Vpr supports HIV-1 infection in CD4^+^ T cells, whilst identifying findings that require validation in physiologically relevant models.

## 1. Introduction

Similarly to all other retroviruses, lentiviruses, including HIV-1, exhibit the prototypical *gag*, *pol*, and *env* genes, encoding the structural, catalytic, and binding elements of their viral progeny, respectively. Additionally, expression of the two essential regulatory proteins Tat and Rev ensures the correct spatiotemporal transcription and production of viral proteins during the viral life cycle. However, a hallmark of HIV-1 and other lentiviruses is the presence of accessory genes (*nef*, *vif*, *vpu*, and *vpx*/*vpr*) [1,2]. These refer to different virulence factors and they are termed “accessory” proteins, given that they have been described as largely dispensable for infection and viral replication in most cell lines but important for full in vivo pathogenicity, high viral loads, and persistence [3]. Although accessory proteins do not exert any known inherent catalytic activity, they collectively excel at counteracting restriction factors (RFs), hijacking metabolic pathways, and mediating immune evasion, consequently contributing towards successful viral replication, spread, and transmission. All this is achieved as a result of their capacity to engage in highly pleiotropic interactions with a diverse range of host cell components [1,4].

One such factor is Vpr: this 14 kDa, 96 aa protein presents an unstructured N-terminal domain followed by three amphipathic α-helices. Vpr is the HIV-1 accessory protein with the highest abundance in the viral particles, as it specifically interacts with the structural peptide p6 and is therefore encapsidated into the assembling viral particle. As a consequence, it is present in de novo infected cells after uncoating and thought to exert multiple biological effects directly thereupon [5,6]. Its tertiary structure is stabilized by a lipophilic core composed of several sterically convergent hydrophobic residues from helices α1 and α3 [7]. These three helical motifs together comprise the contact sites Vpr employs to recruit protein targets, whilst the unstructured domain is involved in the stabilization of various such contacts, as well as the multimerization of this protein in mature virions [7,8]. Despite extensive efforts being directed towards unveiling the precise role(s) Vpr plays in subverting the host’s physiology, no defined clear functions have been found to explain why *vpr* is maintained within the lentiviral genome and why it is apparently essential for the virus in vivo [4]. This stands in contrast to virtually all other viral accessory proteins, which possess defined “main” functions with major measurable phenotypes in certain cell culture systems, despite their multifunctionality. For instance, Vif counteracts APOBEC-family proteins [9], Vpu antagonizes Tetherin [10], and Vpx degrades SAMHD1 [11] and hijacks the Human Silencing Hub (HUSH) complex [12], whilst Nef interferes with CD4, MHC-I, and SERINC5 [13].

Vpr is instead implicated in disrupting multiple cellular processes in a rather nuanced manner, with various unclear biological phenotypes resulting from a large-scale remodeling of the host cell’s protein landscape [5,14]. Although the following sections will discuss the precise means through which Vpr is able to induce the dysregulation of numerous host cell factors, it is essential to clarify the established fact that this phenomenon entails through the redirection of the proteasomal degradation pathway. Under normal circumstances, DDB1, CRL4, and DCAF1 assemble into a E3 ubiquitin ligase complex capable of marking a bevy of proteins for degradation. The substrate specificity of this complex is dictated by the adaptor protein DCAF1, a multifunctional cellular factor with an additional serine/threonine kinase activity canonically involved in the regulation of various processes, such as cell cycle progression, histone modification [15], and telomerase regulation [16]. In the context of HIV-1 infection, however, Vpr has been shown to directly bind DCAF1, earning it the name “Vpr-binding protein” (VprBP). This tripartite interaction is based on the crosstalk between various regions of DCAF1’s β-propeller and Vpr’s α1- and α3-helices, with its unstructured domain further locking this contact in place. By virtue of this, Vpr can significantly influence the manner in which DCAF1 acquires its targets, thereby contributing towards reshaping the intracellular environment in favor of HIV-1 [17].

As such, this interaction is also implied to play a key role in one of Vpr’s most accepted phenotypic functions, that is, to arrest cells in G2. Nevertheless, for G2 arrest itself, neither the functional relevance nor the exact mechanism is clearly established, and compelling evidence for this happening in infected primary CD4^+^ T cells is not widely available; details of the latter will be discussed in the following sections. Indeed, a great deal of confusion in the Vpr field may originate from the fact that a large proportion of data stem from studies employing artificial cell lines, non-relevant models, or in vitro cell-free systems, much thereof not having yet been confirmed in a cellular context. Hence, the main objective of the present review is to summarize Vpr’s known multifaceted activities along various steps of the viral life cycle, emphasizing the evidence supporting these functions in the context of primary CD4^+^ T cells. Importantly, we will explicitly not delve deep into the relevance of Vpr in macrophages, which are the second most relevant type of primary target cell for HIV-1 in vivo.

## 2. Reverse Transcription and Nuclear Delivery of the Pre-Integration Complex

Upon host cell entry, reverse transcription of the viral genome is one crucial event to take place in the replication cycle of HIV. In order to generate a dsDNA proviral genome, the virion-packaged reverse transcriptase (RT) synthesizes a continuous (-)ssDNA strand using the (+)ssRNA viral genome as a template. However, assembling a positive-sensed strand demands the presence of RNA primers and consequently delivers a discontinuous DNA molecule featuring gaps, flaps, and tertiary structures reminiscent of the lagging strand generated during chromosomal DNA replication [18]. Already in this step, Vpr plays an important role, as a 1998 in vitro study (by the term in vitro we refer herein to cell-free systems) demonstrated its capability to sequester Lysyl RNA synthetase (LysRS) [19], thus preventing the aminoacylation of tRNA^Lys3^ that is later packaged into viral particles and serves as a primer for RT to initiate highly processive reverse transcription upon host cell delivery [20].

Patching the plus-strand into a continuous molecule is something the error-prone RT is unable to perform by itself, and Vpr has also been proven to hijack a variety of DNA repair enzymes to compensate for this. A clear example is the Vpr-mediated repurposing of UNG2 and the RPA2 subunit of the DNA damage response (DDR)-associated replication protein A complex, which together lead to the repair of abasic or APOBEC3G-deaminated sites in the said strand, reducing the overall mutation rate of reverse transcription [7,21,22]. Notably, UNG2 is a known interaction partner of the aforementioned DCAF1 protein [7,23,24,25], and to further assist this process, Vpr is thought to simultaneously induce the proteasomal degradation of various host cell factors by virtue of its ability to hijack the CRL4-DDB1-DCAF1 E3 ubiquitin ligase complex [7]; such is the case with Exonuclease 1 (Exo1) and the HLTF helicase, which would otherwise excise newly synthesized proviral DNA and catalyze the reversal and dissociation of the replication fork, respectively [26,27,28].

Concomitantly with this step, it is nowadays accepted that the intact viral capsid containing the PIC ferries its dsDNA payload along the host’s cytoskeleton into the nucleus [29]. Indeed, the protein shell of the pre-integration complex (PIC) was initially thought to act as a protective measure against restriction factors like TRIM5α [30] or MX2 [31]. Other studies, in line with growing evidence of capsid uncoating happening in the nucleus, underscore the fact that the HIV-1 capsid protein lattice prevents the recognition of viral nucleic acids by cytosolic pattern recognition receptors [31]. Vpr itself may also harbor a docking function by interacting with the nuclear pore complex, thereby supporting nuclear capsid translocation [32]. During nuclear entry, architectonical remodeling of the PIC may occur [33], where this complex eventually undergoes uncoating and so allows the preferential integration of the provirus into H3K36me3-rich, actively transcribed chromatin [34]. Aiding the integration process is one of Vpr’s first described functions, and its presence in the nucleus seems crucial for the establishment of a productive infection in CD4^+^ T cells [35]. Despite the fact that it does not possess a classical nuclear localization signal (NLS) in its structure, Vpr nonetheless exhibits a highly karyophilic character, a characteristic attributed to the LxxLL motifs found in α-helices 1 and 3 [36]. Vpr has been demonstrated to bind karyopherin-α in vitro and thereby increase its affinity for basic NLSs such as the ones found in the C-termini of the integrase and matrix proteins of the PIC [37]. By doing so, it would facilitate the FEZ1-mediated acceleration of the PIC’s nuclear import whilst diminishing the likelihood of RFs damaging the PIC [38], although these findings may necessitate refinement in light of the fact that intact viral capsids can enter the nucleus [29].

Given that Vpr is specifically incorporated into assembled viral particles and thus exerts a biological role upon de novo infection, it is clear that the presence of Vpr plays a pivotal role early in the HIV life cycle; not only may it support viral genome integrity before, during, and after reverse transcription, but it may also simultaneously facilitate its journey into the host’s nucleus. Importantly, these findings are conceptually compatible with the current paradigm change, which states that uncoating does not take place directly after viral fusion with the cellular plasma membrane, but rather during or after nuclear import [29,39]. In line with this, Vpr was shown to rapidly traverse to the nucleus after viral entry, although this has thus far only been established in cell lines [40].

What has been proven in primary CD4^+^ T cells is that Vpr degrades the aforementioned host cell factors, i.e., Exo1 and HLTF in a DCAF1-dependent manner [5]; whether Vpr selectively depletes the tRNA pool in infected primary lymphocytes remains to be confirmed, just inasmuch as it does indeed aid the processes of NPC docking of viral capsids and PIC nuclear import. Furthermore, even though Exo1 and HLTF have been shown to be degraded by Vpr in primary T cells, evidence for their functional involvement in G2 cell cycle arrest stems from immortalized cell lines. Factually, this is the most frequent level of experimental evidence available when Vpr functions are biologically connected to their supposed role in inducing G2 arrest, which is discussed in detail in the following section.

## 3. Viral Genome Transcription and G2 Cell Cycle Arrest

The work of Soto and colleagues highlighted how determinant the activation status of CD4^+^ T cells can be towards the outcome of infection; lightly activated subsets are poised to serve as reservoirs for HIV to establish latency upon returning to an inactive phenotype, whereas actively proliferating lymphocytes exhibit a fruitful but short-lived burst in viral production, culminating in apoptosis [41]. Contrastingly, unstimulated lymphocytes were long regarded as inconsequential regarding viral persistence, as HIV disfavors the establishment of infection therein [41,42], although novel data have shed light on the importance this subpopulation exhibits towards latency development. Indeed, naïve CD4^+^ T cells were observed not only to possess unintegrated proviruses in a variety of extrachromosomal configurations [43,44], but the extent of their infection was found to strongly predict reservoir size and diversity, given that this subset can repopulate immune compartments upon depletion of central memory CD4^+^ T cells [45,46]. Unintegrated proviruses could gradually decay and not ever give rise to a productive infection, but state-of-the-art diagnostics underscore the fact that infected naïve lymphocytes do harbor a mixed cohort of defective as well as intact unintegrated proviruses [47], therefore entertaining the feasibility of viremic rebound stemming from this cell population under favorable in vivo conditions [48]. Supporting this hypothesis, Dupont and collaborators recently demonstrated in primary T lymphocytes that Vpr can target the DDR protein SLF2 for degradation, which results in the SMC5/6 complex being unable to compact and repress unintegrated viral DNA intermediates. This would eventually increase the probability of unintegrated viral genomes gaining access to adequate integration sites in the host’s chromatin in a Vpr-dependent manner, eventually allowing for viral transcription and the various physiological effects resulting therefrom [49].

In T lymphocytes with successfully integrated proviruses, engendering a favorable setting for HIV-1 to exhibit optimal transcription is another phenomenon where Vpr might play an important role. Long terminal repeat (LTR) promoters are known for containing diverse *cis*-acting regulatory elements upstream of the proviral CATA box [50], including Sp-, NF-κB-, NFAT-, and AP1-binding sites, along with a downstream Tat-responsive element [51]. Importantly, while these transcriptional factors (TFs) are accepted as the most canonical regulators of the LTR, a recent study in CD4^+^ T cells from patients with low-level viremia identified a plethora of other TFs which directly or indirectly regulate LTR transcriptional activity [52]. About two decades ago, a study in Jurkat T cells postulated a correlation between Vpr’s abilities to transactivate the LTR and cause G2 arrest [53]. Since then, multiple other studies on this topic have been carried out on immortalized cell lines, including T cell-derived models, where it was observed that Vpr-dependent transactivation relies on its capacity to recruit various isoforms of the Sp transcription factor family to the LTR [54,55,56], although this effect proved highly cell type-dependent. Only in 2019 did the work of Hotter and collaborators finally demonstrate the importance of Sp1 availability towards viral expression in the context of CD4^+^ T cells, though the role of Vpr in this process was not assessed in their study [57]. Other work carried out in lymphocytic cell lines indicates that Vpr tethers the glucocorticoid receptor by mimicking the interaction with a steroid receptor coactivator (SRC), which induces it either to localize into the nucleus and into its binding site or to remain complexed in the cytosol in order for PARP-1 to be sequestered, thus preventing the latter from outcompeting Tat on its responsive element. Vpr was also seen to directly bind p300/CBP, itself an SRC proven to potentiate the effects of Tat [55,58]. However, again, these host cell interactions have never been functionally connected to G2 arrest in primary T cells.

As has been mentioned, it is generally hypothesized that the cell cycle plays a determinant role in retroviral physiology, as its arrest at the G2/M checkpoint seems to foster a cellular milieu optimal for HIV gene expression, which is suggested by the observation of increased LTR transcriptional activity in G2-arrested, HIV-infected Jurkat T cells [59]. Amongst other potential mechanisms, this phenomenon might be attributed to the likely presence of an internal ribosome entry site (IRES) in the 5′ leader sequence of viral RNA transcripts, since canonical cap-dependent translation is known to wane in favor of IRES-dependent initiation during the G2/M checkpoint [60]. Cell cycle checkpoint activation traditionally ensues as a consequence of DNA damage in the form of single- or double-strand breaks (SSBs and DSBs, respectively), base pair mismatches, stuck replicative machinery, or other types of lesions requiring structural amends. Nuclear sentinel proteins such as ATR or ATM are recruited to the damaged sites, where they phosphorylate various targets involved in cell cycle checkpoint control, in turn causing not only the upregulation of genes encoding DNA repair machinery but also the inactivation of cyclin-dependent kinases. This eventually results in cell cycle arrest, granting the cell time for repairs to take place or steering it towards apoptosis in case the damage is too extensive [61].

The specific involvement of Vpr in promoting G2 cell cycle arrest is one of its most studied properties, as it is one of the few Vpr phenotypes that can easily be recapitulated in transfected or infected cell lines, including immortalized CD4^+^ T cells [61]. Furthermore, induction of G2 arrest by Vpr can be attributed to its interaction with the CRL4-DDB1-DCAF1 E3 ubiquitin ligase complex, and this interaction is disrupted by specific mutations in Vpr (see the following section) [62]. On the other hand, induction of G2 arrest by Vpr has long been characterized as having a cytostatic effect on infected CD4^+^ T cell populations and leading to apoptosis [63], which is difficult to reconcile with Vpr possessing an important role during the early phase of the viral life cycle. The Bieniasz group recently demonstrated that Vpr can elicit the proteolysis of the chromosome periphery protein CCDC137 in primary lymphocytes, which could trigger an increase in γH2AX-rich nuclear foci as a consequence of DDR pathway engagement and thus lead to an interruption in cell cycle progression [64]. Another recent study in primary T cells shows that Vpr-triggered DNA damage responses also prevent the repressive TF ZBTB2 from coupling with the LTR-binding protein ZASC1, an interaction which would otherwise cause the recruitment of histone deacetylases (HDACs) and the subsequent silencing of the chromatin surrounding the proviral integration site [65]. Of note, G2 arrest in primary CD4^+^ T cells was not assessed in this study.

The U2OS [66], HT1080, and MIT-23 [67] cell models have been employed to demonstrate that Vpr expression is sufficient to induce DSBs and stall DNA replication, activating chromatin damage markers and leading to ATR recruitment. Independently thereof, Vpr was also able to inhibit both the homologous recombination (HR) and non-homologous end joining (NHEJ) pathways of DNA repair, contributing towards the genomic instability of the infected cell [66]. Evidence from a 2014 study suggests that the former pathway could be specifically jeopardized due to the Vpr-DCAF1-PLK1 axis hijacking the scaffolding agent SLX4com, causing the untimely activation of the EME1 and MUS81 nucleases and eventually contributing towards G2 arrest via the dismantlement of active replication forks whilst simultaneously disabling the resolution of DNA ultrafine anaphase bridges, further destabilizing the host genome [68]; again, this seemingly seminal finding still awaits confirmation in primary T cells. Meanwhile, the targeted proteasomal degradation of SIRT7, whose HDAC activity is of paramount importance in the repair of DSBs and the maintenance of DNA repair sites, was used to explain how Vpr compromises the NHEJ repair pathway in another recent study by Zhou et al. on HEK293T cells [69]. Finally, HIP1 was recently identified as yet another interaction partner of Vpr which showed a positive effect on G2 arrest irrespective of DSB induction, indicating that alternative mechanisms may be responsible for the stalling of the cell cycle and thus opening many potential research avenues in this particular field. However, this last finding was observed in the context of macrophages and immortalized cell lines, warranting confirmation in T lymphocytes as well [70].

Likewise, Vpr has been implicated in disrupting multiple other structures crucial for cell cycle progression, including the anaphase promoting complex/cyclosome, the centrosome, the replisome, as well as chromosomal centromeres and telomeres. Barbosa and colleagues recently showcased in CD4^+^ T cells how transmitter/founder Vpr variants coopt the DCAF1-DDB1 E3 Ligase complex to target APC1 for proteasomal degradation, potentially disabling the vital role it plays in the metaphase-to-anaphase transition [71]. Park and colleagues’ work on primary T cells evidenced that a similar mechanism is followed by Vpr in subverting centrosome activity, sequestering the kinase PLK4 and leading to centriole duplication [72]. Analogously, Wang and colleagues demonstrated that TERT, the catalytic subunit of telomerases, is also a target for Vpr-mediated proteolysis in HIV-1-infected CD4^+^ T lymphocytes [73]. Together, these effects destabilize the genomic integrity of the host and could perhaps be linked to a higher incidence of neoplasia in HIV patients [74]. Additionally, Vpr was shown to degrade the cell cycle-associated helicase activator MCM10, thereby stalling the DNA-unwinding machinery and cell cycle progression. Further, Vpr’s association with DCAF1 was implicated in the degradation of centromeric protein A (CENP-A), potentially contributing towards cell cycle delay and genomic instability. These findings, however, were exclusively observed in immortalized cell lines and must undergo further validation [75,76].

In summary, even though Vpr-mediated induction of G2 arrest in T cells is implied to result in a higher transcriptional state of the HIV-1 LTR, evidence for this causality happening in primary CD4^+^ T cells is lacking. Conversely, Vpr phenotypes associated with G2 in cell lines could also converge with regulating RT and integration in primary CD4^+^ T cells. Such a mechanism would explain the finding that a bevy of proteins playing a role in the regulation of the DDR are degraded by Vpr via the CRL4-DDB1-DCAF1 E3 ubiquitin ligase complex. Some recent developments in this field have seen the confirmation of various Vpr functions in the context of CD4^+^ T cells, including its indirect role in antagonizing ZBTB2-ZASC1 LTR silencing, as well as the targeted degradation of SLF2, CCDC137, TERT, and APC1, together with the sequestration of PLK4. Other compelling pieces of evidence, such as the directed degradation of SIRT7, HIP1, MCM10, and CENP-A, in addition to the Vpr-mediated sequestration of SLX4com to engage DNA damage response and trigger G2 arrest, still warrant validation in CD4^+^ T cells to attain full physiological relevance.

**Figure 1:**
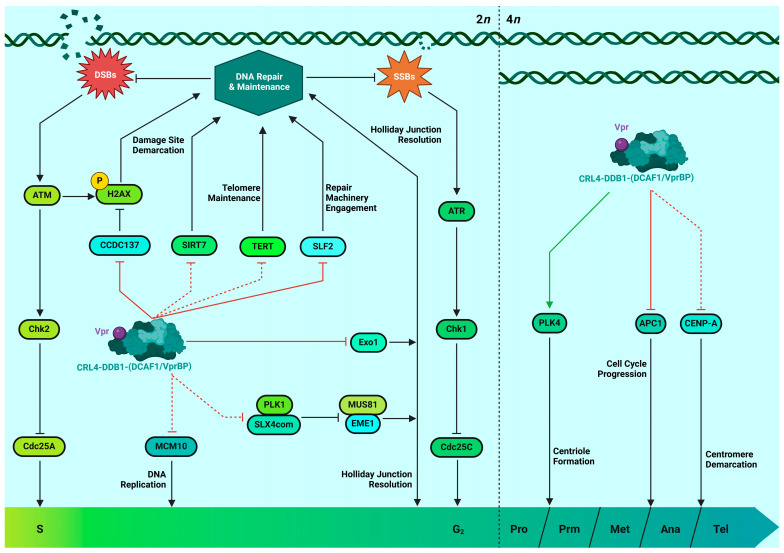
Scheme illustrating Vpr’s involvement in G2 arrest induction and DNA damage response engagement. Inhibitory/deleterious associations, resulting in target protein blocking/degradation, are depicted as red blunt-ended lines. Activating/beneficial associations are depicted as green arrows. Associations confirmed in CD4^+^ T cells or physiologically relevant models (i.e., ex vivo cultured human lymphoid tissue, patient-derived samples, etc.) are depicted as full arrows, whereas dotted arrows illustrate associations yet to be characterized therein. Pro = prophase; Prm = prometaphase; Met = metaphase; Ana = anaphase; Tel = telophase. (Figure created with BioRender).

In total, surprisingly few studies have been able to establish a link between Vpr and G2 arrest occurring in primary CD4^+^ T cells. For one, the Planelles group showed that HIV-1-infected activated primary T lymphocytes exhibit a G2/M phenotype that is partially dependent on ATM/ATR and Vpr [61,63,77,78,79]. Second, a study in humanized mice described Vpr-induced G2/M arrest in regulatory T cells undergoing depletion before strongly sustaining HIV-1 replication [80]. Hence, while these studies implicate Vpr’s G2 arresting capacity in vivo, it is essential and of very high relevance to carefully delineate the detailed context and relevance of Vpr-induced G2/M arrest during primary CD4^+^ T cell infection. To facilitate this and recapitulate the complex and different order of events governing Vpr-mediated G2 arrest, an overview of Vpr’s involvement in this phenomenon and DDR engagement is depicted in Figure 1.

## 4. Systems-Level Manipulation of the Host Cell

In recent years, several studies have sought to assess the overall systems-level manipulation Vpr exerts on the host cell, hinted at by its ability to degrade a bevy of host cell factors, resulting in major changes of the host cell proteome and transcriptome. A study by Malim’s group demonstrated how HIV-1 utilizes virion-delivered Vpr to induce wide-ranging transcriptional alterations in primary CD4^+^ T cells as early as 4.5 h post-infection, clearly anticipating interferon-stimulated gene (ISG)-dependent expression changes by about 8 h [81]. Another study in Jurkat T cells indicated that the presence of Vpr suffices to upregulate the expression of miRNA 210-5p, itself eliciting the downmodulation of TGF-β-response repressor TGIF2. This protein is involved in proliferation and cell differentiation, meaning its depletion may indirectly also contribute towards G2 arrest [82]. In line with this finding, not only did Sato and collaborators observe enhanced cell cycle arrest in Treg cells in HIV-1 infected humanized mice, but they also demonstrated that this subset’s higher proliferative potential renders it more permissive towards productive infection of CCR5-tropic viruses, followed by large-scale apoptosis and the subsequent depletion of this lymphocyte subpopulation [80]. This phenomenon could partly explain the higher incidence of autoimmune disorders reported in HIV patients, as this regulatory T cell subset secretes TGF-β to help modulate immune responses and temper otherwise uncontrolled inflammation to commensurate proportions [83].

Tightly coupled to transcription, splicing is another phenomenon over which HIV has been demonstrated to exert a very significant influence [84,85]. The first in vitro evidence for the involvement of Vpr in this process appeared in 2005, where cellular pre-mRNA splicing was shown to be severely inhibited [86]. Shortly afterwards, a second in vitro study revealed that Vpr is able to hijack several components of the spliceosome, such as SAP145, U1-70K, U2AF65, and U2B’’, as well as U1 and U2; this led not only to the inhibition of splicing of cellular mRNAs but also enabled preferential processing of viral primary transcripts [87]. Vpr’s capability to regulate splicing of viral transcripts was later mapped to its N-terminus, as tagging this site led to oversplicing and ablated viral production in SupT1 cells [88].

In comparison to other viral pathogens, HIV-1 is notorious for its flexibility regarding ribosome recruitment strategies. As touched upon in the previous section, IRES-dependent initiation could be favored during G2 cell cycle arrest to aid viral expression [60]. However, a recent study indicated that the IRES present in the Gag-coding region showcases inefficient usage in the context of infected CD4^+^ T cells, with ribosome profiling identifying concomitant translation of upstream open reading frames and viral genes, significantly reducing the translation efficiency of the latter [89]. This appears to be greatly alleviated by the presence of the translation-associated RNA helicase DDX3 [90], whose pharmacological inhibition led to apoptosis in latency-reversed CD4^+^ T cells from combined antiretroviral therapy (cART)-treated HIV^+^ donors [89,91]. Further, a 2012 in vitro study correlated Vpr’s capability to induce G2 arrest with the phospho-4E-binding protein (P-4E-BP)-mediated sequestration of eIF4E, explaining the overall decline in the translation of cellular mRNAs observed upon infection; whilst fully spliced viral mRNAs continued to be detected in the vicinity of ribosomes at the 5′-cap, partially or non-spliced transcripts were instead predominantly associated with the stress-related nuclear cap-binding complex (CBC) [92,93]. This finding was recently confirmed in HIV-infected CD4^+^ T cells, where it became clear that the non-canonical CBC-to-CBP80 cap exchange in unspliced viral transcripts was enabled by the hypermethylation of the 5′-cap. Ultimately, this phenomenon allows HIV to escape the Vpr-elicited eIF4E-dependent translational bottleneck, permitting sustained viral protein production via an emergency cap-exchange mechanism despite global translational shutdown. However, the authors did not establish a direct link to Vpr in this study [94].

Immune function is one of the main aspects of lymphocyte physiology deeply affected by Vpr upon infection. An innovative study by Chiodi’s group performed mass cytometry on CD4^+^ T cells from HIV^+^ donors, showing a negative correlation between the timing of cART administration and the preservation of immune function; this was reflected by the abundance of surface markers associated with inflammation, cell migration, and survival [95]. Shan et al. identified that CD4^+^ lymphocytes undergoing an effector-to-memory transition inadvertently facilitate the establishment of HIV-1 latency, particularly of CXCR4-tropic strains. Their results indicated that this population’s initial transcriptional pattern renders them permissive to infection, but prolonged viral gene expression cannot be sustained, since the phenotypic switch to memory T cells favors quiescence [96]. This phenomenon was further explored by Jolly’s group, who further characterized the HIV-mediated immunological reprogramming of infected T cells. Their work identified that viral spread within lymphocyte populations via cell-to-cell contacts preferentially leads to infection of resting memory T cells. In this scenario, virion-delivered Vpr induces the establishment of a tissue-resident phenotype by synergizing with IL-7 to activate STAT5, ultimately inducing a virus-favorable transcriptional program [97]. Ectopic STAT5 activation has recently been observed to alter the epigenome of CD4^+^ T cells and push their phenotype towards polyfunctionality, although such an alteration may present yet unidentified implications in the context of infection, perhaps playing a significant role in HIV pathogenesis [98]. However, this effect does appear to synergize with the presence of other leukocytes like non-cytolytic CD8^+^ T cells [99] or activated macrophages [100], which have been linked with the establishment of HIV latency by pushing CD4^+^ T cell metabolism, signaling, and transcription towards quiescence.

Overall, and as was introduced earlier, Vpr’s capability to hijack the CRL4-DDB1-DCAF1 E3 ubiquitin ligase complex allows HIV to directly modify the host cell’s proteome upon infection, underpinning the establishment of an optimal intracellular milieu perhaps even before proviral integration and its subsequent transcriptional and translational effects. Lehner’s group recapitulated on Vpr’s capability to direct a vast array of targets towards proteasomal degradation using CEM-T4 T cells, showing how the overwhelming majority of observed alterations is mostly attributable to Vpr [14]. In parallel, they backed this up by analyzing synchronously infected primary CD4^+^ T cells, resulting in a detailed overview of HIV-elicited proteomic changes at 24 and 48 h post-infection. Indeed, results in primary CD4^+^ T cells not only mirrored the broad depletion pattern of Vpr in CEM-T4 T cells but further confirmed the targeted degradation of several proteins previously only studied in immortalized cell lines, such as HLTF, UNG2, as well as the DDR endonucleases MUS81 and EME1 [101]. Together, these groundbreaking studies present virion-delivered Vpr as the main driving force behind the proteomic remodeling elicited by HIV in the context of infection.

In summary, the evidence gathered in a physiologically relevant context indicates that Vpr induces an early uptick in ISG transcription upon infection [81] and indirectly enables cap hypermethylation, allowing viral mRNAs to overcome the host’s transcriptional blockage [92,93,94]. It has also been shown that Vpr plays a leading role in manipulating IL-7 and STAT5 immune signaling, prompting the establishment of latency with the help of the host lymphocyte’s cellular environment [97]. In addition, the CRL4-DDB1-DCAF1-mediated remodeling of the host proteome not only factually explains many of the alterations reported in this cell population but also confirms that Vpr actively targets the HR-related proteins MUS81 and EME1 for degradation, endorsing a branch of the hypothesis that attributes Vpr to the DNA damage response pathway [101]. On the other hand, and despite much evidence existing to support the notion that Vpr subverts the spliceosome to aid viral processing, its supporting facts remain to be validated in CD4^+^ T-cells. This also holds true for the Vpr-induced upregulation of miR-210-5p; despite intriguingly tying TGF-β and NF-κB signaling into the Vpr-induced G2 arrest phenomenon, it has thus far only been observed in immortalized cell lines and thus requires further validation in T lymphocytes.

## 5. Signaling Disruption and Immune Evasion

Vpr is thought to modulate various signaling pathways, many of them intrinsically involved in immune activation, inflammation, proliferation, and survival [102,103,104]. Amongst them, the NF-kB signaling cascade has long been suspected of playing a central role. However, comprehending how the activation and nuclear translocation of NF-κB is influenced by Vpr remains an undeciphered riddle, largely due to the puzzling nature of the available evidence in this field. A 1997 study on PBMCs implicated Vpr in suppressing NF-κB-mediated gene activation [105], an effect later observed in CD4^+^ T cells, albeit without establishing a direct connection to Vpr [100]. This link was first proven to ensue via DCAF1 in 2013 [106] and then thoroughly explained by Khan et al. in 2020, who showed how virion-delivered Vpr associates with DCAF1 in order to interact with subunit 1 of karyopherin-α (KPNA1), thus sabotaging the nuclear import of NF-κB in CD4^+^ T cells [107]. Another study examined the NF-κB-inhibiting capability of multiple Vpr alleles in PBMCs, the results indicating that only de novo-synthetized Vpr from simian immunodeficiency viruses SIVcol and SIVolc retains this trait; in the case of HIV-1, this function is seemingly delegated to Vpu instead [108]. 

Antithetically, a relatively large body of evidence supports the notion that both the canonical as well as the non-canonical pathways of NF-κB activation are directly triggered by Vpr to varying extents. Such is the case with the Vpr-induced phosphorylation of the kinase TAK1, proven to result in the nuclear translocation of NF-κB and AP-1 in PBMCs via the canonical pathway [109]. In Jurkat T cells, a similar mechanism is followed by Vpr in order to phosphorylate IKKα/β, activating both pathways in parallel for an enhanced NF-κB response [110]. Specifically, activating NF-κB via the non-canonical pathway has recently been described to aid HIV latency reversal [111], demonstrated in CD4^+^ T cells from healthy donors [112]. On the other hand, the “atypical” pathway of NF-κB activation is known to be triggered by genotoxic stress and may play a role in HIV pathogenesis [113]. Liang and collaborators hinted at a link existing between Vpr-mediated cell cycle arrest and NF-κB translocation [114], with a novel study performed in macrophages indicating that Vpr-triggered DNA damage activates ATM, followed by the nuclear transport of various TFs, including NF-κB, CEBPB, and JUN [115]. Vpr’s capacity to interact with the SRC p300/CBP, until now only described in immortalized cell lines, did not only aid Tat’s transactivating role but was also accompanied by a significant increase in binding affinity between NF-κB and its LTR regulatory sequence [55,58].

NF-κB activity proves relevant even when HIV-1 is pitted against antiviral defenses, as might be the case with PYHIN-mediated innate immunity. Kirchhoff’s group demonstrated in CD4^+^ T cells that various members of this restriction factor family [116] antagonize HIV transcriptional activity by sequestering Sp1 [57,117], with their newest results indicating that an additional NF-κB binding site in the LTR of subtype C members enables sustained transcription and enhanced reactivation potential, a notion that could explain the overrepresentation of this HIV-1 clade at the global scale [118]. Given that NF-κB regulates inflammation and the induction of ISGs but is simultaneously essential for proliferation and survival as well activating the HIV-1 LTR, its manipulation represents a double-edged sword in the context of HIV infection. As such, it warrants a highly precise timing for the virus to exploit its stimulatory boost whilst escaping subsequent deleterious effects. All in all, the reigning theory in this field describes NF-κB activation as being required during the early stages of the viral life cycle to transcriptionally engage the proviral LTR, but its translocation is inhibited at later stages via diverse mechanisms, thereby lowering the expression of genes associated with antiviral innate immunity [119]. Nevertheless, it remains an enigmatic phenomenon whether Vpr activates or suppresses NF-κB differentially across the viral life cycle, as there is a large body of conflicting data related to this topic, even in primary CD4^+^ T cells.

Apart from NF-κB, Vpr influences other transcription factors involved in the regulation of lymphocytic physiology and regulation of the HIV-1 LTR. The nuclear factor of activated T cells (NFAT), of paramount importance for T cell activation, proliferation, differentiation, and survival, is canonically activated by membrane receptors coupled to Ca^2+^ mobilization, resulting in its nuclear translocation. Using a variety of cellular models as well as primary lymphocytes, our group demonstrated that virion-delivered Vpr is able to bypass upstream stimulation to activate NFAT and thus support productive HIV infection, a phenotype that is additionally correlated with Vpr-mediated LTR transactivation and G2 arrest in T cell lines [120]. Closely associated with innate immune activation and inflammation, interferon regulatory factor 3 (IRF3) is another signaling intermediate acting downstream of TLR activation. This protein has been shown to undergo proteasomal degradation via the presence of both Vpr and Vif in Jurkat T cells and PBMCs [121]. Further, the possibility of IRF3 localizing to the nucleus is negated by Vpr’s ability to sequester and degrade KPNA1, a phenomenon demonstrated in CD4^+^ T cells [107].

Besides rewiring intracellular communications to seize control over the host, HIV-1 also employs a multipronged approach to circumvent most defensive mechanisms placed against it. Though all of its accessory proteins participate in this task to different extents [122], Vpr might possess a thus far unprecedented role therein, due to its immediate deployment directly upon host cell entry as well as its unique ability to induce the degradation of target proteins. For example, the interaction between Vpr and SLX4com through VprBP, implicated in eliciting G2 arrest, was also shown to induce the processing of excess HIV-1 DNA, preventing its sensing and the consequent type 1 IFN response. This, however, has only been shown in HeLa cells thus far [68]. APOBEC3G, one of the most potent antiretroviral restriction factors identified to date [123], was originally thought to be exclusively counteracted by Vif, although novel evidence from a study in the MT4 and H9 T cell lines suggests that Vpr can initially perform this task before de novo production of Vif ensues [124]. LAPTM5, a restriction factor that reroutes HIV-1 gp120 for lysosomal degradation in macrophages, has been recently observed to be a target of Vpr via DCAF1; this was additionally observed in LAPTM5-retransfected CD4^+^ T-cells, confirming its interaction with Vpr. Whilst intriguing, the physiological relevance of this finding to T cells remains enigmatic, as this restriction factor is normally not present in the lymphoid proteome [125].

Silencing, both at the transcriptional and epigenetic levels, is another antiviral mechanism that HIV-1 can override with the help of Vpr. SRSF1, an IFN-modulated splicing factor, was observed to hinder overall LTR activity in Jurkat and THP-1 cells [126]. In this context, a recent pioneering study involving direct RNA nanopore sequencing in CD4^+^ T cells identified the Vpr-mediated upregulation of ciTRAN, an HIV-modulating circular RNA that sequesters SRSF1 to prevent it from burdening viral gene expression [127]. Further, our group employed primary T lymphocytes to demonstrate that, after aiding proviral integration, PHF13 is robustly degraded by Vpr in order for HIV to escape its restrictive effects on transcription [128]. Of note, PHF13 might regulate major epigenetic DNA modifications [129,130], and its degradation by HIV-1 Vpr is thus highly reminiscent of HIV-2/SIV Vpx-mediated degradation of the HUSH complex [12]. Other mechanisms to counter epigenetic silencing by Vpr happen through the targeted proteasomal degradation of various nucleosome remodeling and deacetylase (NuRD) complex components. This was first observed in the HEK293T and HeLa cell lines, where Vpr coopted DCAF1 to degrade ZIP and sZIP, two DNA-binding adaptors known to interact with NuRD [131]. Later, a Vpr-dependent degradation of the scaffolding protein CTIP2 was reported in CD4^+^ T cells, thereby negating the NuRD-mediated recruitment of HDACs, lysine demethylases, and histone methyltransferases to the LTR [132]. Finally, the recently discovered putative RNA ploymerase-binding factor RPRD2 [133] is able to restrict HIV-1 by binding viral nucleic acids and recruiting the epigenetic repressors HUSH [134] and PAF1 [135] to deposit silencing marks therein. Though only so far observed in macrophages and HeLa CD4^+^ cells, McKnight’s group demonstrated that Vpr targets RPRD2 for degradation via CRL4-DDB1-DCAF1, protecting the provirus from silencing and boosting overall transcription rates in the affected cell [136,137].

Both the timing Vpr exhibits in re-orchestrating intracellular signaling and its swift defusal of pre-deployed antiviral defenses clearly indicate that the strategy followed by HIV is inherently aimed towards preventing the establishment of a widespread antiviral response while triggering several pathways that generate a cellular milieu favorable for viral replication. So far, the available evidence pertaining to CD4^+^ T cells paints Vpr as a central mediator in the induction of early NFAT signaling that is later on sustained by Nef [138,139]. Induction of ISGs and the interferon response is ablated by interfering with IRF3 nuclear translocation. NF-κB signaling is manipulated both negatively by sabotaging its nuclear translocation together with DCAF1 and KPNA1 as well as positively by triggering its non-canonical and atypical activatory pathways. Whether Vpr is also able to trigger NF-κB’s canonical activation pathway and the DNA damage response is thereby activated warrants further study in T lymphocytes, along with the characterization of the timepoint at which enhanced NF-κB signaling stops being favored by HIV. Likewise, Vpr has been confirmed to degrade LAPTM5, PHF13, and CTIP2, while upregulating ciTRAN, helping HIV evade transcriptional and epigenetic repression in primary T lymphocytes. RPRD2 and APOBEC3G degradation, albeit highly interesting, have yet to be studied in primary CD4^+^ T cells.

## 6. Inflammation and Apoptosis

Heavily intertwined with both signaling and immunity, inflammation and apoptosis are essential aspects of cellular physiology that could be affected by Vpr [140]. In the year 2000, Roux and collaborators used primary CD4^+^ T cells to demonstrate that Vpr elicits the expression of IL-8 and TNF-α upon infection [141], with a 2015 study further confirming not only the release of the latter but also mechanistically implicating the previously mentioned DDB1 and TAK1 proteins therein [142]. Notably, the aforementioned proinflammatory transcriptional program induced by Vpr at the systems level [81] seems to be at least partly dependent on Vpr enhancing cGAS-mediated viral DNA sensing, boosting innate immunity and inflammation [143]. While this may initially appear counterintuitive, the same study demonstrated that Vpu negatively regulates this effect at later stages of infection. Therefore, a certain level of proinflammatory activity by Vpr could prove beneficial for viral replication, or alternatively arise as a “side effect” of its biological activity. Further, a recent genome-wide association study performed on PBMCs from a broad cohort of HIV^+^ patients unraveled that infected cell populations exhibit an epigenetic dysregulation in distinct gene clusters; ISG promoters of patients with higher viral loads presented a hypomethylation pattern, which translated into an enhanced proinflammatory response. In parallel, uncontrolled infection was also found to correlate with the hypermethylation of cell markers associated with the commitment to a T follicular helper cell fate, a lymphocyte subset essential for the sustainment of long-term immunity and the development of specialized humoral responses [144]. Though not assessed by this study, it is plausible that Vpr could play a significant role in the early establishment of an epigenetic landscape favoring viral proliferation, as evidenced by its capacity to selectively manipulate mediators of chromatin remodeling, such as PHF13 and CTIP2 [128,132].

Programmed cell death is frequently the endgame of inflammatory responses, as well as one of the leading mechanisms behind lymphocyte depletion in HIV^+^ patients, a hallmark of retroviral pathophysiology. Vpr’s inherence therein has long been observed, but the precise mechanism whereby it leads to cell death remains a highly controversial topic. Virion-associated Vpr has been demonstrated to induce cytotoxicity by itself whilst also potentiating the effect of Fas-mediated apoptosis in activated PBMCs [145]. Conti and collaborators observed that, at early timepoints post-infection, Vpr confers on infected PBMCs protection from apoptosis despite the presence of TNF-α signaling, although a completely opposite outcome presented itself at later timepoints [146]. In 2002, a study in PBMCs and primary T lymphocytes demonstrated that Vpr permeabilizes the mitochondrial membrane and induces the release of proapoptotic proteins towards the cytosol, triggering Caspase 9-mediated cell death [147]. This effect was later attributed to the Vpr-mediated activation of the apoptosis regulator Bax and to the DCAF1-mediated degradation of mitofusin 2 (Mfn2), both in T cell-derived models as well as in primary T lymphocytes [77,148]. In light of the fact that Bax is a known effector of inflammation and apoptosis [149] acting downstream of the DNA damage response [150], a mechanistic correlation between the Vpr-triggered induction of cell death and G2 checkpoint arrest became increasingly debated, with Sakai and colleagues proposing such a link existing not only for Vpr but independently also for Vif [151].

However, the initiation of programmed cell death in CD4^+^ T cells is also dependent on multiple other factors, including the microenvironment a cell may encounter itself in. Trinité et al. demonstrated in primary T lymphocytes how the proapoptotic effect of virion-delivered Vpr can be counteracted by the activity of common γ-chain cytokines like IL-7 and IL-4, frequently found in the sites of active HIV-1 replication [152]. Detected in the serum of infected patients [153,154], virion-free Vpr has also been suggested to permeate into T cells and thus elicit a diverse array of effects depending on the identity of the target. In lymph nodes, such an effect may trigger the death of uninfected bystander T cells [155] through both the intrinsic and extrinsic pathways; indeed, Cohen’s group demonstrated that soluble recombinant Vpr entering bystander T-cells can directly rouse the G2 arrest-inducing DNA damage response whilst simultaneously upregulating the NKG2D ligands ULBP-1 and ULBP-2 [156,157]. Analogously, Vassena et al. observed how this Vpr-triggered DDR also leads to the upregulation of poliovirus receptor (PVR) on the surface of T lymphocytes, increasing the likelihood to trigger NK-mediated cytotoxicity [158]. Other studies showed that both IL-6 and soluble Vpr stimulate viral reactivation in latently infected cell lines through the ROS-induced TLR4-MyD88 signaling axis, leading to further IL-6 release and the subsequent expansion of the reactivating stimulus [154]. Tying into this hypothesis, a recent study in macrophages unveiled that Vpr can selectively deplete the methylcytosine dioxygenase TET2 via CRL4-DDB1-DCAF1, negatively impacting 5mC levels in the IL-6 promoter and likely leading to sustained IL-6 production and release, greatly fueling the reactivation of latent HIV-1 in adjacent T cells [159].

In a nutshell, HIV appears to ensure its spread across critical leukocytic populations by promoting constant inflammation, thereby sensitizing cells for productive infection, whilst remaining unnoticed by immune surveillance; in cells harboring latent provirus, such an environment can trigger viral reemergence and production. It has so far been shown in CD4^+^ T cells that Vpr degrades TAK1 to boost TNF-α and IL-8 signaling, while simultaneously activating mitochondrial Bax and degrading Mfn2 to initiate apoptosis. Recombinant Vpr has also been shown to permeate bystander lymphocytes, upregulating markers that increase their likelihood of undergoing cytotoxic cell death. Future research has yet to unveil if the targeted degradation of TET2 observed in macrophages holds true in CD4^+^ T cells, as well as if this protein provides infected cells protection from apoptosis under specific conditions, if at all. Additionally, it remains an open research avenue to identify whether Vpr represents a nexus between G2 arrest induction, DNA damage responses, and cell death.

## 7. Variation in Vpr’s Primary Structure

Multiple studies have sought to map the functional properties of Vpr down to the amino acid level by employing various naturally occurring and culture-derived variants, together with site-directed mutagenesis. These efforts have resulted in the identification of several amino acid positions that play a vital role in defining the functional properties of Vpr’s structural elements, eventually granting it the ability to interact with diverse partner proteins as well as to effectively localize to certain subcellular compartments in order to exert many of its hallmark effects. Of central importance to Vpr’s pathogenicity and functionality, glutamine 65 is located in a short leucine-rich motif within the α3 helix, a domain responsible for several Vpr attributes, including nuclear localization, cell cycle arrest, and multimerization. The work of Wu et al. demonstrated that Q65 is critical for stabilizing the Vpr–DCAF1 interaction, as this residue partakes in the formation of a hydrophobic pocket where Trp1156 of DCAF1 docks whilst concomitantly engaging in hydrogen bonding with Ser1136 of the latter’s WD40 β-propeller domain [7]. The Q65R substitution, detected in long-term non-progressor HIV patients, renders Vpr unable to interact with DCAF1 [160], as experimentally confirmed by Skowronski’s group in ex vivo-infected CD4^+^ T cells [26]. Given how crucial the takeover of the said E3 ligase adaptor is to Vpr’s broad-acting effects in the host cell, it comes as no surprise that this mutant is associated with a milder course of infection, in addition to the fact that it fails to induce cell cycle arrest, cannot localize to the nuclear envelope, and does not present any alteration in cytopathic potential [160].

Cytopathicity, in turn, is another extensively studied Vpr function. Soares and collaborators first identified a R77Q mutation, together with additional Q3R substitution, in a long-term non-progressor pediatric patient who showed no symptoms other than recurrent ear infections [161]. Ever since, multiple studies have collaboratively mapped cytopathic potential to a Vpr moiety known as the mitochondrial membrane permeabilization (MMP)-inducing sequence, which contains two H(F/S)RIG motifs along the amino acid stretch 72–80. These motifs, observed to bring about gross cell enlargement and replication arrest [5,162,163], were more closely studied in ex vivo-cultured human lymphoid tissue. This work unraveled the physiological importance of residues R77 and R80, since point mutations R77A, R77Q, and R80A all led to reduced viral replication, whilst the latter two mutants also showed an impairment in the induction of T cell depletion [164]. Markedly, the R80A mutation possesses a dominant-negative phenotype in the context of co-expression with wt Vpr, disabling its capability to trigger G2 arrest. This phenotype is reverted when Vpr presents both R80A and Q65R mutations, further underscoring the link between Vpr’s G2 arrest-inducing and DCAF1-recruiting abilities [61,62,160] A recent structural study by Byeon et al. demonstrated that helix α3 of Vpr represents an important contact interface both for DCAF1 as well as for the DNA repair protein hHR23A; this finding links proteasomal degradation and ubiquitin recognition with the nucleotide excision repair pathway and G2 arrest, potentially explaining the phenotypes engendered by mutations on residues R77 and R80 [165].

Other physiologically relevant sites are Vpr’s hydrophobic amino acids, not only responsible for stabilizing its inner core [5] but also having been observed to affect the spatial configuration of its α-helices, influencing Vpr’s capability to localize to the nucleus as well as to oligomerize at the nuclear envelope; such is the case with positions I63, L67, I70, and I74 [166]. In addition, hydrophobic positions in helix α1, such as W18 and L22, have been described as crucial to oligomerization, whilst potentially also contributing towards G2 arrest and cytopathicity [167]. Further, S79 has been identified to be a target for phosphorylation [168], this step seemingly being necessary for the cell cycle arresting activity of Vpr [169]. Thus far, the only mutations associated with rapid disease progression are the relatively uncommon R36W, L68M, and R85Y, the first also being characterized as eliciting increased viral replication by granting Vpr a higher oligomerization potential [170]; this evidence notwithstanding, the mechanisms whereby such an effect takes place remain unelucidated. All these mutations are part of a long list of single sequence polymorphisms experimentally associated with a variety of phenotypes, which, despite greatly contributing to a better understanding of Vpr’s functional properties, have so far only been studied in immortalized cell lines and/or are not necessarily naturally occurring. As such, their study in physiologically relevant models as well as their further characterization at the molecular level represent an open research avenue. A complete summary of these findings can be found in Table 1.

## 8. Concluding Remarks

The present work has evidenced that great advances have been achieved in elucidating the role that Vpr plays during HIV-1 infection. It has become clear that this accessory protein is a virulence determinant for HIV-1 to achieve full in vivo pathogenicity, transmission, spread, and cellular persistence. Considering that it is abundantly present within viral particles, its direct intracellular delivery is one of the first events to take place during the HIV life cycle, representing an optimal timepoint for its multifaceted effects to execute the furtive domination of infected lymphocytes.

**Figure 2:**
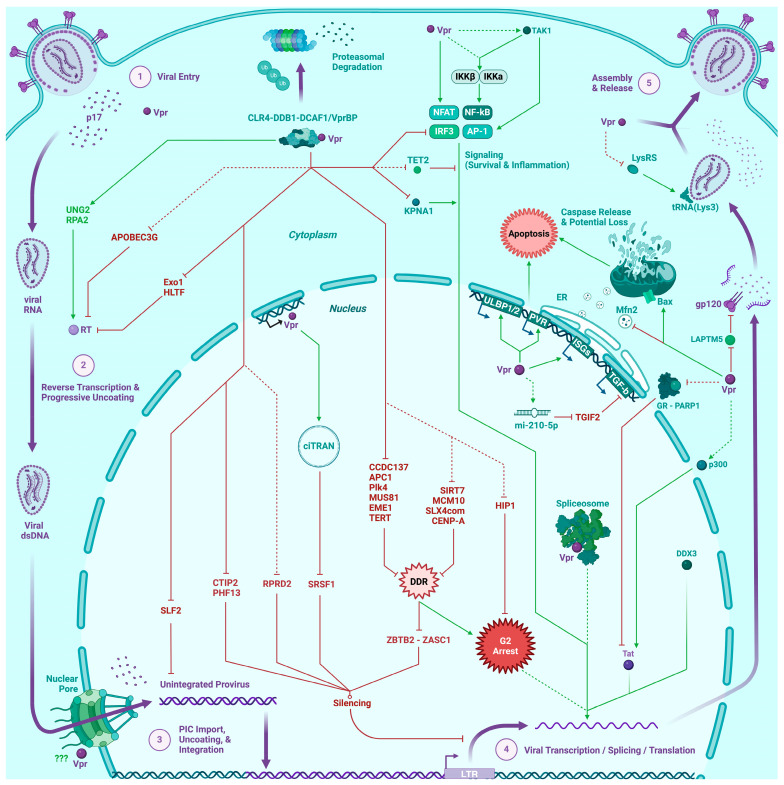
Scheme illustrating Vpr activities. Viral processes (numbered text, arrows) and components (objects) are depicted in purple. Host cell processes (text) and components (objects) are depicted in teal. Inhibitory/deleterious associations are depicted as red objects or blunt-ended lines. Activating/beneficial associations are depicted as green arrows. Associations confirmed in primary CD4^+^ T cells or physiologically relevant models (i.e., ex vivo cultured human lymphoid tissue, patient-derived samples) are depicted as full arrows, while dotted arrows illustrate associations yet to be characterized therein. (Figure created with BioRender).

Given the compelling evidence that has been gathered regarding Vpr’s functionality in the context of primary T cells and presented within this review (summarized in Figure 2), we postulate that Vpr’s proinflammatory and activating nature sensitizes newly infected as well as bystander T cells for productive infection. Concomitantly, it exploits a repertoire of mechanisms to manipulate host cell factors involved in organizing the cell cycle and DNA repair, further aiding reverse transcription and viral integration. As these processes are intertwined with the activation of antiviral defenses, including innate immunity and the interferon response, Vpr has likely also evolved mechanisms to counteract the detrimental effects induced by its own proviral activities. Of course, this model warrants experimental validation in addition to the evidence detailed herein. Additionally, various existing pieces of the complex Vpr puzzle await their final validation in CD4^+^ T lymphocytes (see Figure 1 and Figure 2). As such, the detailed spatiotemporal characterization of the mechanisms encompassing Vpr-mediated G2 arrest, as well as their functional interconnection with essential steps of viral replication, will undoubtedly serve as the necessary precedent to attain a more exhaustive understanding of Vpr’s role in mediating viral pathogenicity, taking special consideration of the particularities various CD4^+^ T cell subsets exhibit during the course of HIV-1 infection.

## Figures and Tables

**Table 1:** Summary of Vpr mutations with an altered phenotype and/or clinical relevance. HAND = HIV-associated neurocognitive disorder; LTNP = long-term non-progressor; RP = rapid progressor; C-ter = carboxyl-terminus; N-ter = amino-terminus; α1 = alpha-helix 1; α2 = alpha-helix 2; α3 = alpha-helix 3, HLT = human lymphoid tissue.

Mutation	Domain	Phenotype	Clinical Association	Characterized In	Reference
Q3R	N-ter	Lower viral production (in tandem with R77Q) Lower cytopathic potential	LTNP	HeLa *S. cerevisiae* Patient PBMCs CD4^+^ T cells	[161,171,172,173]
P5A	N-ter	Reduced oligomerization Reduced transactivation	None	CemM7, Jurkat HeLa CD4^+^ T cells Macrophages	[120]
P10A	N-ter	Reduced NFAT activation Reduced transactivation	None	CemM7, Jurkat HeLa CD4^+^ T cells Macrophages	[120]
P14A	N-ter	Reduced transactivation Diminished G2 arrest	None	CemM7, Jurkat HeLa CD4^+^ T cells Macrophages	[120]
N16H	N-ter	No change with respect to WT	LTNP	Patient PBMCs	[174]
W18A/S/E/R	α1	Altered virion packaging and delivery Diminished G2 arrest Lower cytopathic potential	None	Jurkat *S. pombe.*	[167,175]
T19A	α1	No change with respect to WT	LTNP	Jurkat, HeLa COS-7 PBMCs	[170]
E21/24Q	α1	Altered subcellular localization Reduced NFAT activation No oligomerization No PARP1 translocation No transactivation	None	CemM7, Jurkat HeLa CD4^+^ T cells Macrophages	[120]
L22A/S/E	α1	Altered subcellular localization Altered virion packaging and delivery Diminished G2 arrest No oligomerization (A) Reduced PARP1 translocation (A) Increased GRE activation (A) Increased transactivation (A) Nuclear aggregation (A)	None	CemM7, Jurkat HeLa, CV1 HEK293 CD4^+^ T cells Macrophages	[120,167,176,177,178]
L23F/A	α1	Altered subcellular localization No oligomerization Altered virion packaging and delivery No PARP1 translocation Diminished G2 arrest Diminished interaction with 14-3-3η Abolished interaction with hCGI	None	CemM7, Jurkat HeLa, CV1 COS-7, MT4 CD4^+^ T cells Macrophages *S. cerevisiae* HEK293	[120,171,173,176,179,180,181,182,183]
E24G/Q	α1	Lower cytopathic potential Impaired interaction with UNG2 Diminished G2 arrest Altered Na^+^ permeability	None	*S. pombe* Hippocampal neurons CD4^+^ T cells	[175,184,185,186]
E25K	α1	Lower cytopathic potential Altered virion packaging and delivery Altered subcellular localization Impaired interaction with UNG2 Diminished G2 arrest Resistance to Fumagilin	None	Jurkat *S. pombe* *S. cerevisiae* COS-7, MT4 HEK293, HeLa	[58,173,175,180,184,187,188,189,190]
K27M	α1	Aggregation Loss of G2 arrest Lower cytopathic potential Reduced oligomerization Reduced NFAT activation Reduced transactivation No PARP1 translocation Altered subcellular localization Abolished interaction with hCG1 No induction of TNF-α	None	CemM7, Jurkat J-Lat, HEK293 HeLa, MT4C5 CD4^+^ T cells Macrophages	[120,142,163,183]
A30L/P/F/S	α1	Loss of G2 arrest No oligomerization Altered virion packaging and delivery Altered subcellular localization Unable to interact with nucleoporins No Iκκα stimulation	None	HEK293, COS-7 HeLa, RD Jurkat, CV1 SupT1, MT4 *S. cerevisiae*	[32,110,173,180,183,190,191,192,193,194,195,196,197,198]
P35A	α1-α2 loop	Altered subcellular localization Altered virion packaging and delivery No oligomerization Diminished G2 arrest Lower cytopathic potential Reduced NFAT activation Reduced PARP1 translocation Reduced transactivation Reduced interaction with CypA	None	CemM7, Jurkat HeLa, HEK293 CD4^+^ T cells Macrophages Structural studies	[120,182,191,199,200,201]
R36W	α1-α2 loop	Aggregation Altered virion packaging and delivery Increased oligomerization Increased viral replication Diminished G2 arrest	RP	Jurkat, HeLa HEK293, COS PBMCs	[170,176,191]
L39G	α2	Altered virion packaging and delivery Altered subcellular localization	None	HeLa, CV1 HEK293	[176]
G41N/A55	α2	High neurocognitive deficit	HAND	Patient PBMCs	[202]
G41N/N28S	α2	Inability to sequester APC1	None	CD4^+^ T cells Macrophages	[71]
G41S/I37	α2	Low neurocognitive deficit	HAND	Patient PBMCs	[202]
L42A/G	α2	Reduced GRE activation Altered virion packaging and delivery Diminished G2 arrest	None	HeLa, CV1 HEK293	[176]
H46W	α2	Altered subcellular localization (in tandem with H45W)	None	CD4^+^ T cells	[35]
Y50A	α2	Diminished G2 arrest	None	HeLa, Jurkat	[163]
W54R	α3	Lower cytopathic potential Impaired interaction with UNG2 Impaired interaction with SMUG Increased viral mutation rates Abolished interaction with hCG1 Impaired IgA class switch reccombination	None	HEK293, HeLa MT4C5, J-Lat CH12F3-2, HT-29 MAGIC-5B *S. pombe* CD4^+^ T cells Dendritic cells Structural studies	[7,21,23,25,76,142,158,183,187,203,204,205,206,207,208,209,210,211]
V57L	α3	Altered virion packaging and delivery Altered subcellular localization	None	MT4	[198]
A59P	α3	Altered virion packaging and delivery Altered subcellular localization Loss of G2 arrest	None	HeLa, RD Jurkat, CV1	[193,194,195]
R62P/N/D	α3	Diminished G2 arrest Unable to form nuclear foci Altered subcellular localization Diminished interaction with DCAF1	None	*S. cerevisiae* HEK293 MT4C5, J-Lat CD4^+^ T cells Structural studies	[7,142,173,177,212]
I63E/K/F/T	α3	Lower cytopathic potential Diminished G2 arrest Reduced oligomerization Altered virion packaging and delivery Altered subcellular localization (in tandem with L64R)	None	Jurkat, SupT1 COS-7, MT4	[166,171,173,180,198]
L64P/A/S	α3	Unable to interact with DCAF1 Altered virion packaging and delivery Altered subcellular localization Loss of G2 arrest Loss of cytopathic potential No oligomerization No PARP1 translocation No NFAT activation No GR activation No transactivation Increased Vpr turnover (P)	None	CemM7, Jurkat HeLa, CV1, RD CD4^+^ T cells Macrophages HEK293, TZM-bl	[120,176,177,178,194,210,213,214,215,216]
64-68A	α3	Altered subcellular localization Reduced oligomerization No PARP1 translocation No NFAT activation No transactivation No virion incorporation Loss of G2 arrest Loss of cytopathic potential	None	CemM7, Jurkat HeLa CD4^+^ T cells Macrophages	[120]
Q65R	α3	Unable to interact with DCAF1 Altered subcellular localization Unable to form nuclear foci Reduced oligomerization Loss of G2 arrest Loss of cytopathic potential Diminished viral replication Reduced induction of TNF-α Reduced vDNA nuclear import Diminished accumulation of γ-H2AX Increased Vpr turnover	LTNP	HEK293, MT4 CEM.SS, HeLa MT4C5, J-Lat CD4^+^ T cells Patient PBMCs Macrophages Dendritic cells Structural studies	[5,26,142,160,163,198,204,210,217,218,219,220,221,222,223,224,225,226,227]
L67E/A/S/P	α3	Diminished G2 arrest Lower cytopathic potential Altered subcellular localization Altered virion packaging and delivery Reduced oligomerization Reduced GRE activation Abrogated splicing takeover (P) No transactivation	None	Jurkat, HeLa SupT1, CV1, RD HEK293, TZM-bl	[35,166,171,176,177,179,194,195,228,229,230,231]
L68M/A/S	α3	Reduced oligomerization Lower viral production Diminished G2 arrest Altered subcellular localization Reduced vDNA nuclear import (A)	RP	TZM-bl Jurkat, HeLa COS, CV1 HEK293, RD PBMCs	[170,176,177,192,215,232]
I70S	α3	Altered subcellular localization Reduced oligomerization	None	Jurkat, SupT1	[166]
H71R/C/Y/A	α3	Diminished G2 arrest No oligomerization Altered virion packaging and delivery Lower cytopathic potential Altered subcellular localization Diminished viral replication Impaired interaction with UNG2 Diminished interaction with DCAF1 Diminished interaction with Cdc25C Increased NF-κB stimulation (Y) Increased viral mutation rates Diminished accumulation of γ-H2AX (A)	None	HEK293, HeLa RD, Jurkat CV1, TZM-bl *S. pombe* CD4^+^ T cells Dendritic cells	[24,25,59,79,175,186,187,192,193,194,195,204,207,209,233,234]
F72L/A	α3	Altered subcellular localization Diminished G2 arrest (A) (in tandem with R73A)	LTNP	Patient PBMCs	[235,236]
R73A/S	α3	Diminished interaction with hHR23A No transactivation	None	Jurkat, HEK293 Structural studies	[58,165]
I74E	α3	Diminished G2 arrest Lower cytopathic potential	None	Jurkat, SupT1	[166]
G75A	α3	No oligomerization Altered virion packaging and delivery Altered subcellular localization Diminished G2 arrest	None	HEK293, HeLa RD, Jurkat CV1	[191,193,194,195]
C76A/S	α3	Diminished interaction with DCAF1 No oligomerization No NFAT activation Lower cytopathic potential Loss of G2 arrest Reduced PARP1 translocation Reduced transactivation Altered virion packaging and delivery	None	HEK293 CemM7, Jurkat HeLa, RD CD4^+^ T cells Macrophages	[120,194,195,233,237]
R77Q	α3	Lower viral production Lower cytopathic potential Loss of G2 arrest Reduced PARP1 translocation No Iκκα stimulation	LTNP	CemM7, Jurkat HeLa, J-Lat MT4C5, HEK293 *S. cerevisiae* CD4^+^ T cells Macrophages Patient PBMCs	[110,120,160,164,173]
R77A	α3	Lower viral production Reduced PARP1 translocation	LTNP	CemM7, Jurkat HeLa CD4^+^ T cells Macrophages Ex vivo HLT	[120,164]
S79A/I	C-ter	Lack of phosphorylation Loss of G2 arrest Increased interaction with UNG2 No transactivation (in tandem with R80K) No induction of TNF-α No Iκκβ stimulation Abolished 14-3-3θ association to targets Altered subcellular localization	None	J-Lat, MT4C5, CV1 Jurkat, PBMCs HEK293, HeLa *S. cerevisiae* Macrophages CD4^+^ T cells	[58,110,142,163,168,169,173,187,193,210,238]
R80A	C-ter	Lower viral production Loss of G2 arrest No NFAT activation No induction of TNF-α No transactivation Diminished viral replication Diminished interaction with hHR23A Reduction in whole-cell ubiquitination Abolished interaction with 14-3-3η Abolished 14-3-3θ association to targets Unable to form nuclear foci Unable to activate unintegrated provirus Reduced interaction with CypA Reduced vDNA nuclear import Diminished accumulation of γ-H2AX	LTNP	SupT1, MT4 CemM7, CEMx174 Jurkat, HeLa J-Lat, MT4C5 HEK293, TZM-bl *S. cerevisiae* CD4^+^ T cells Macrophages Ex vivo HLT Structural studies	[23,26,58,79,120,142,158,164,165,166,169,173,177,181,183,191,196,198,200,210,212,216,217,218,221,225,226,236,238]
R85Y/P	C-ter	Altered subcellular localization Lower viral production Increased oligomerization Diminished G2 arrest	RP	Jurkat, HeLa, COS PBMCs, HEK293	[170,239]
R90N	C-ter	No change with respect to WT	LTNP	Jurkat, HeLa, COS PBMCs	[170]
R90K	C-ter	Suppressed IL-12 production Lower cytopathic potential Loss of G2 arrest Reduced transactivation Impaired IgA class switch recombination	LTNP	HEK293, HeLa CH12F3-2 Jurkat *S. pombe* Dendritic cells	[23,59,171,175,183,187,203,211,240]
